# Meta-analysis confirms association between *TNFA-*G238A variant and JIA, and between *PTPN22*-C1858T variant and oligoarticular, RF-polyarticular and RF-positive polyarticular JIA

**DOI:** 10.1186/1546-0096-11-40

**Published:** 2013-10-25

**Authors:** Merlyn J Kaalla, K Alaine Broadaway, Mina Rohani-Pichavant, Karen N Conneely, April Whiting, Lori Ponder, David T Okou, Sheila Angeles-Han, Kelly Rouster-Stevens, Milton R Brown, Larry B Vogler, Lynn B Jorde, John F Bohnsack, Michael P Epstein, Sampath Prahalad

**Affiliations:** 1 Departments of Pediatrics, Emory University School of Medicine, Atlanta, GA, USA; 2 Department of Human Genetics, Emory University School of Medicine, Atlanta, GA, USA; 3 Department of Pediatrics, University of Utah School of Medicine, Salt Lake City, UT, USA; 4 Children’s Healthcare of Atlanta, Atlanta, GA, USA; 5 Eccles Institute of Human Genetics, University of Utah, Salt Lake City, UT, USA

**Keywords:** Genetics, Juvenile idiopathic arthritis, Association, Replication

## Abstract

**Background:**

Although more than 100 non-HLA variants have been tested for associations with juvenile idiopathic arthritis (JIA) in candidate gene studies, only a few have been replicated. We sought to replicate reported associations of single nucleotide polymorphisms (SNPs) in the *PTPN22*, *TNFA* and *MIF* genes in a well-characterized cohort of children with JIA.

**Methods:**

We genotyped and analyzed 4 SNPs in 3 genes: *PTPN22 C1858T* (rs2476601), *TNFA* G-*308A*, *G-238A* (rs1800629, rs361525) and *MIF G-173C* (rs755622) in 647 JIA cases and 751 healthy controls. We tested for association between each variant and JIA as well as JIA subtypes. We adjusted for multiple testing using permutation procedures. We also performed a meta-analysis that combined our results with published results from JIA association studies.

**Results:**

While the *PTPN22* variant showed only modest association with JIA (OR = 1.29, *p* = 0.0309), it demonstrated a stronger association with the RF-positive polyarticular JIA subtype (OR = 2.12, *p* = 0.0041). The *MIF* variant was not associated with the JIA as a whole or with any subtype. The *TNFA-238A* variant was associated with JIA as a whole (OR 0.66, *p* = 0.0265), and demonstrated a stronger association with oligoarticular JIA (OR 0.33, *p* = 0.0006) that was significant after correction for multiple testing. *TNFA-308A* was not associated with JIA, but was nominally associated with systemic JIA (OR = 0.33, *p* = 0.0089) and enthesitis-related JIA (OR = 0.40, *p* = 0.0144). Meta-analyses confirmed significant associations between JIA and *PTPN22* (OR 1.44, *p* <0.0001) and *TNFA-238A* (OR 0.69, *p* < 0.0086) variants. Subtype meta-analyses of the *PTPN22* variant revealed associations between RF-positive, RF-negative, and oligoarticular JIA, that remained significant after multiple hypothesis correction (*p* < 0.0005, *p =* 0.0007, and *p <* 0.0005, respectively).

**Conclusions:**

We have confirmed associations between JIA and *PTPN22* and *TNFA* G-*308A.* By performing subtype analyses, we discovered a statistically-significant association between the *TNFA-238A* variant and oligoarticular JIA. Our meta-analyses confirm the associations between *TNFA-238A* and JIA, and show that *PTPN22 C1858T* is associated with JIA as well as with RF-positive, RF-negative and oligoarticular JIA.

## Background

Juvenile idiopathic arthritis (JIA) refers to a collection of chronic autoimmune arthropathies in children. Although the etiology of JIA is complex, substantial evidence supports the importance of genetic factors in susceptibility to JIA [1-3]. While associations between JIA and variants in HLA are well established, non-HLA genetic variants also play a role in JIA susceptibility, and have increasingly been identified by genome-wide and candidate gene studies [4-6]. However, candidate-gene association studies of non-HLA variants in JIA have led to inconsistent results. Of nearly 100 non-HLA polymorphisms tested for associations with JIA by candidate gene studies, only a handful of associations have been replicated in independent cohorts [2,5,7,8]. More recently, the International JIA Immunochip consortium has confirmed many of the genetic associations and also identified several new loci with genome-wide evidence for association [9].

In previous non-HLA genetic association studies, a functional variant at *PTPN22* (C1858T) has been consistently associated with JIA [6,10-12]. Variants at *MIF* (G-173C) and *TNFA* (G-308A, G-238A) genes have also been associated with JIA, although some of the studies show mixed results [13-21]. The reasons for non-replication are myriad, but chief among them is inadequate power due to small cohorts. One way to overcome this limitation is to perform meta-analyses of published studies. Meta-analyses have confirmed associations between JIA and genetic variants in *STAT4, TNFAIP3, IL2RA,* and *CCR5*, and have failed to find an association with CTLA4 variants [7,22,23]. Our objectives were to test previously associated variants at the *TNFA* and *MIF* loci in an independent JIA cohort and then to combine these results with published results in a meta-analysis. Furthermore, since prior studies of the *PTPN22* variant have investigated combined JIA cohorts, we sought to investigate associations between JIA categories and the *PTPN22* variant by meta-analysis.

## Methods

Cases were 647 children with JIA from Pediatric Rheumatology clinics at the University of Utah (N = 437 cases, 750 controls) and Emory University (N = 210 cases). Patients were diagnosed according to the ILAR criteria [24]. The median age of onset was 5.8 years, and 67% of the cases were female. There were 50 children with systemic JIA, 48 with rheumatoid factor (RF)-positive polyarticular JIA, 159 with RF-negative polyarticular JIA, 58 with enthesitis-related arthritis (ERA), 287 with oligoarticular JIA, and 45 with other categories. Controls were 751 healthy adults (59% female) screened for several common autoimmune diseases and ascertained from the same geographic region as the Utah cases. Only subjects of self-reported Northern European ancestry were included in this study. A questionnaire was used to screen controls for autoimmune disorders. Controls who reported an autoimmune disorder were excluded. Subjects were enrolled under protocols approved by the Institutional Review Boards at the University of Utah and Emory University.

### Genotyping

DNA was isolated from peripheral blood using the Puregene DNA purification kit from Qiagen (Valencia, CA). Subjects were genotyped for four SNPs in three loci: rs2476601 (C1858T) in the *PTPN22* locus, rs1800629 (G-308A) and rs361525 (G-238A) in the *TNFA* locus and rs755622 (G-173C) in the *MIF* locus. These variants were chosen because of their reported associations with JIA in more than one cohort based on a review of published literature [2,6,10-12,14,16-18,25-31]. Genotyping of cases and controls was performed using Taqman pre-designed SNP genotyping assays (Applied Biosystems, Foster City, CA) according to the manufacturer’s protocols. To ensure quality control, ~3% of the samples were genotyped in duplicate to ensure accuracy and were found to be concordant. DNA samples with low genotyping success were removed from analyses.

### Statistical analysis

Prior to association analysis, we first tested whether each variant was in Hardy-Weinberg equilibrium (HWE) in controls. We then tested for association between each variant and disease outcome using a logistic regression model assuming an additive model of allelic effect and adjusting for gender. From such models, we calculated allelic odds ratios (OR) and 95% confidence intervals (95% CI) that were adjusted for gender. Since these variants have been previously implicated in susceptibility to JIA and other autoimmune disorders, we considered JIA to be the primary disease phenotype. However, since JIA is a collection of several heterogeneous subtypes, we repeated the analyses for specific JIA subtypes based on ILAR criteria. Since each of these variants has been previously implicated as being associated with JIA, we initially focused on nominal significance (*p* < 0.05) but subsequently adjusted for multiple comparisons using a permutation procedure. Our permutation procedure generated 1000 datasets under the null hypothesis of no association between JIA and genotype by repeatedly shuffling the vector of genotypes for each subject in the dataset. This type of permutation procedure preserves both the linkage disequilibrium among variants and the known effects of gender on JIA. We carried out all analyses using the R programming language [32].

As we studied variants previously investigated for association with JIA, we performed a meta- analysis of these variants that combined our study with published case-control association studies of JIA (identified using a PUBMED search). Using allele frequency data derived from these studies, we performed meta-analyses of *PTPN22, TNFA* and *MIF* associations with JIA using a fixed-effects model that weighted studies by number of subjects. We established significance using Cochran-Mantel-Haenszel tests. We used Cochran’s Q test to assess heterogeneity among studies, with a significance level set at *p* < 0.10, as is recommended for Cochran’s Q test [33,34]. When we identified heterogeneity between studies, we repeated the analyses after removing the study responsible for the heterogeneity. As an alternate analysis, we performed all the meta-analyses using an unconditional generalized linear mixed-effects model, which allowed for random study effects. We performed the meta- analyses using the R package “metafor” [35].

## Results

We found that all four SNPs were in approximate HWE in our controls, using the Bonferroni-adjusted alpha threshold of 0.0125. In our cohort, we removed 1 of the 647 cases missing gender information. Furthermore, we found that 17 individuals were missing genotype data at *MIF* genotypes, 28 were missing *PTPN22*, 10 were missing *TNFA238*, and 40 were missing *TNFA308* yielding genotyping success rates ranging between 97.2% to 99.3%.

Using logistic regression, we observed that the *PTPN22* 1858 T variant showed a nominal association in our JIA cohort (OR = 1.29, *p* = 0.0309) (Table 1). After stratification by subtype, we observed the variant was nominally associated with two subgroups: those with RF- positive polyarticular JIA (OR 2.12; *p* = 0.0041) and oligoarticular JIA (OR = 1.35, *p* = 0.0400) (Table 2).

**Table 1:** Case-control analysis of PTPN22, TNFA and MIF variants and JIA

	**Cases**		**Controls**			
**Variant**	**# Cases**	**MAF**	**# Controls**	**MAF**	**OR (95% CI)**	***p-value***
*PTPN22 C1858T*	636	0.13	733	0.11	1.29 (1.02-1.62)	**0.0309**
*MIFG-173C*	638	0.18	742	0.17	1.06 (0.88-1.29)	0.5353
*TNFA G-238A*	638	0.04	749	0.06	0.66 (0.46, 0.95)	**0.0265**
*TNFA G-308A*	628	0.14	729	0.17	0.82 (0.66, 1.01)	0.0574

**Table 2:** Results of case-control association of PTPN22, MIF and TNFA variants among JIA sub-phenotypes

	***PTPN22***		***MIF***		***TNFA G-238A***		***TNFA G-308A***	
**Subtype**	**# Cases**	**OR (95% CI)**	**# Cases**	**OR (95% CI)**	**# Cases**	**OR (95% CI)**	**# Cases**	**OR (95% CI)**
Systemic	50	1.06 (0.53, 1.91)	49	1.53 (0.93, 2.45)	50	1.69 (0.76, 3.36)	50	**0.33 (0.13, 0.69)**^**4**^
RF-Positive	48	**2.12 (1.24, 3.49)**^**1**^	47	1.06 (0.60, 1.75)	48	*	47	0.98 (0.54, 1.66)
RF-Negative	154	1.09 (0.74, 1.57)	158	0.86 (0.61, 1.19)	157	1.14 (0.67, 1.86)	156	0.85 (0.60, 1.18)
ERA	55	1.09 (0.55, 1.97)	56	1.23 (0.74, 1.98)	56	0.94 (0.35, 2.06)	56	**0.40 (0.18, 0.78)**^**5**^
Oligoarticular	284	**1.35 (1.01, 1.80)**^**2**^	283	1.06 (0.83, 1.36)	284	**0.33 (0.16, 0.59)**^**3**^	276	0.96 (0.73, 1.25)

1 *p* = 0.0041; 2 *p* = 0.0400; 3 *p* = 0.0006; 4 *p* = 0.0089; 5 *p* = 0.0144; Statistically significant results are bolded. * Represents an analysis that was not conducted due to small cell size.

**Figure 1:**
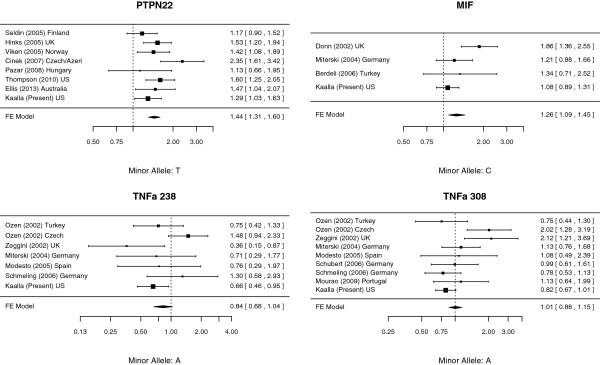
Forest Plots of meta-analyses, including all studies.

The *PTPN22* variant has been investigated for an association with JIA in seven other case-control comparisons (Table 3) [6,8,10-12,25,26]. (Some of our subjects were included in a replication study of several autoimmunity associated variants in JIA [6]. In order to avoid duplication, the replication cohort used in the study by Thompson et al. was not included in our meta-analysis). Subjects in the studies included in our meta-analysis were of European ancestry. The pooled meta-analysis confirmed a strong association between JIA and *PTPN22* with an OR of 1.44 (95% CI [1.31, 1.60]), *p* < 0.0001 (Figure 1, Table 4). When all seven studies plus our data were included, Cochran’s Q test for heterogeneity was not significant at the *p* = 0.10 threshold (*p* = 0.11).

**Table 3:** Studies included in meta-analyses

					**Cases**		**Controls**	
**Gene**	**Author**	**Ref number**	**Country**	**Publication year**	**# Cases**	**MAF**	**# Controls**	**MAF**
*PTPN22 C1858T*	Seldin et al.	[7]	Finland	2005	230	0.18	1400	0.15
	Hinks et al.	[5]	UK	2005	661	0.15	595	0.10
	Cinek et al.	[18]	Czech/Azeri	2007	130	0.21	400	0.10
	Pazar et al.	[19]	Hungary	2008	150	0.09	200	0.08
	Thompson et al.	[4]	USA	2010	809	0.14	531	0.09
	Ellis	[29]	Australian	2013	324	0.10	568	0.07
	Viken et al.	[6]	Norway	2005	320	0.16	555	0.12
	Kaalla et al.	Present	USA		637	0.13	733	0.11
*MIF G-173C*	Donn et al.*	**[**[16]**]**	**UK**	**2002**	**526**	**0.19**	**259**	**0.11**
	Miterski et al.	[9]	Germany	2004	150	0.24	390	0.21
	Berdeli et al.	[13]	Turkey	2006	67	0.13	153	0.10
	Kaalla et al.	Present	USA		639	0.18	742	0.17
*TNFA G-238A*	Ozen et al.*	**[**[22]**]**	**Turkey**	**2002**	**51**	**0.22**	**93**	**0.27**
	Ozen et al.*	**[**[22]**]**	**Czech**	**2002**	**159**	**0.23**	**100**	**0.17**
	Zeggini et al.	[23]	UK	2002	137	0.03	76	0.09
	Miterski et al.	[9]	Germany	2004	130	0.02	375	0.03
	Modesto et al.	[20]	Spain	2005	55	0.07	59	0.09
	Schmeling et al.	[11]	Germany	2006	228	0.03	196	0.03
	Kaalla et al.	Present	USA		639	0.04	749	0.06
*TNFA G-308A*	Ozen et al.*	[22]	**Turkey**	**2002**	**51**	**0.25**	**93**	**0.31**
	Ozen et al.*	[22]	**Czech**	**2002**	**159**	**0.27**	**100**	**0.16**
	Zeggini et al.	[23]	UK	2002	138	0.24	75	0.13
	Miterski et al.	[9]	Germany	2004	122	0.17	312	0.16
	Modesto et al.	[20]	Spain	2005	55	0.13	59	0.12
	Schubert et al.	[12]	Germany	2006	86	0.15	270	0.15
	Schmeling et al.	[11]	Germany	2006	228	0.14	196	0.17
	Mourao et al.	[21]	Portugal	2009	115	0.12	118	0.11
	Kaalla et al.	Present	USA		629	0.14	729	0.17

*Bolded studies were removed from analysis due to evidence for heterogeneity, using Cochran’s Q test (*p* < 0.10 threshold).

**Table 4:** Meta-analysis results for PTPN22, MIF and TNFA variants and JIA

		**Cases**		**Controls**				
**Gene**		**# Cases**	**MAF**	**# Controls**	**MAF**	**OR (95% CI)**	***p-value***	**Data sources**
*PTPN22 C1858T*	All Studies	3261	0.14	4982	0.11	**1.44 (1.31, 1.60)**	**<0.0001**	4-7, 18,19, 29
*MIF G-173C*	Including Donn et al. [16]	1382	0.19	1544	0.17	**1.26 (1.09, 1.45)**	**0.0356**	9, 13, 16
	Removing Donn et al.	856	0.19	1285	0.18	1.12 (0.96, 1.32)	0.1548	
*TNFA G-238A*	Including Ozen et al. [22]	1399	0.07	1648	0.07	0.84 (0.68, 1.04)	0.1033	9, 11, 20, 22, 23
	Removing Ozen et al.	1189	0.03	1455	0.04	**0.69 (0.52, 0.91)**	**0.0086**	
*TNFA G-308A*	Including Ozen et al. [22]	1583	0.17	1952	0.16	1.01 (0.88, 1.15)	0.925	9, 11, 12, 20-23
	Removing Ozen et al.	1373	0.15	1759	0.16	0.95 (0.83, 1.10)	0.5137	

Of the seven studies, only Hinks et al. study reported data by subtype [10]. We performed a pooled subtype meta-analysis using our data and data from Hinks et al. The meta-analysis confirmed findings from our cohort: the variant was significantly associated with RF-positive, RF-negative, and oligoarticular JIA (*p* < 0.0005, *p* = 0.0007 and *p <* 0.0005, respectively) (Table 5). Findings remained significant after correction for multiple hypothesis testing. Systemic JIA and ERA were not significantly associated with *PTPN22*.

**Table 5:** **Meta-Analysis results for *PTPN22 C1858T *** **subtypes: combining Hinks et al. [**[5]**] data with data from our cohort**

	**Hinks et al. [**[5]**]**			**Kaalla et al. (Present)**			**Combined**	
**Category**	**Size**	**MAF**	**OR (95% CI)**	**Size**	**MAF**	**OR (95% CI)**	**OR (95% CI)**	***p-value***
Systemic	118	0.09	0.89 (0.55, 1.44)	50	0.11	1.05 (0.55, 2.01)	0.94 (0.64, 1.39)	0.7675
RF-Positive	35	0.17	1.79 (0.94, 3.43)	48	0.21	2.24 (1.33, 3.77)	**2.05 (1.37, 3.77)**	**<0.0005**
RF-Negative	135	0.2	2.12 (1.49, 3.02)	154	0.12	1.13 (0.77, 1.66)	**1.56 (1.21, 2.02)**	**0.0007**
ERA	48	0.14	1.36 (0.74, 2.51)	55	0.11	1.04 (0.56, 1.94)	1.19 (0.77, 1.84)	0.4431
Oligoarticular	276	0.15	1.56 (1.16, 2.10)	284	0.14	1.36 (1.01, 1.82)	**1.45 (1.18, 1.79)**	**<0.0005**
Controls	595	0.1	NA	733	0.11	NA	NA	NA

*PTPN22* allele frequency and odds ratios from Hinks et al. [5] and Kaalla et al. (Present) divided by five JIA categories. No covariates were included in the analyses. Pooled data significant at the α = 0.05 threshold level is bolded.

We observed no association between the *MIF* variant in our JIA cohort as a whole (OR 1.06, 95% CI [0.88-1.29], nor after stratification by subtype (Tables 1 and 2). The *MIF* variant has been investigated for an association with JIA in three other case-control comparisons, all of which examined European populations (Table 3) [14,18,31]. The meta-analysis of the three previous studies and our data demonstrated (Figure 1) an association between JIA and *MIF*G-173C (OR 1.26, 95% CI [1.09, 1.45]; *p* = 0.0014). Cochran’s test for heterogeneity was significant (*p* = 0.04), however, because the Donn et al. study including subjects from the United Kingdom [21] demonstrated a much stronger association between *MIF* and JIA than the other studies (Table 3). After removing the study by Donn et al. from the analysis, we found the resulting association between MIF and JIA to be negligible (OR 1.12, 95% CI [0.96, 1.32], *p* = 0.15) (Table 4), suggesting that the initial association was driven by the Donn et al. study. Since the Hinks et al. [10] and Zeggini et al. [30] studies represent work from the same Manchester group, we were concerned that these studies may also lead to heterogeneity in our meta-analyses. Therefore, we tested for effect of the *PTPN22* and the *TNFA* variants, and excluded the Hinks and Zeggini results. Although the magnitude of the odds ratios for the three tests decreased with the reduced sample, inference for all three associations did not change. Additionally, we were concerned that removing apparently heterogeneous studies was too conservative an approach; therefore, we re-ran meta-analyses using a generalized linear mixed-effects model with random study effects. Results of the mixed-effects models can be seen in the Additional file 1: Table S1. Results were comparable to the fixed-effects models in which all studies were included in analysis.

*TNFA*-238A was associated with JIA in our cohort (OR 0.66, *p* = 0.0265) (Table 1). Upon stratifying the analysis by JIA subtype, we observed the most pronounced association between *TNFA*-238A and oligoarticular JIA (OR 0.33, *p* = 0.0006); no other subtypes were significantly associated with this SNP (Table 2). The association between oligoarticular JIA and *TNFA*-238A was the strongest association found in this study and remained significant after adjusting for multiple hypothesis testing using permutation resampling (corrected *p* = 0.0113). While the *TNFA-308A* showed no association with the entire JIA cohort, there was a nominal association between this SNP and systemic JIA (OR 0.33, *p* = 0.0089) and ERA (OR 0.40, *p* = 0.0144) (Tables 1 and 2).

Five studies have previously investigated the *TNFAG-238A* variant [14,16,27,29,30] and seven have investigated the *TNFAG-308A* variant [14,16,17,27-30] (Table 3). One of the studies, by Ozen et al. [29], investigated both *TNFA* variants in Turkish and Czech cases and controls; the others studied Western European populations [29]. The minor allele frequencies (MAF) reported in Ozen et al. differed widely from the other studies: MAF for *TNFAG-238A* in Ozen et al.’s sample was 0.27 (vs. 0.03 for other studies) and MAF was 0.31 (vs. 0.11) for *TNFA G-308A* (Table 3). Including data from Ozen et al. in the meta-analysis (Figure 1) resulted in significant evidence for heterogeneity (*p* = 0.05 and *p* = 0.004 for *TNFAG-238A* and *TNFAG-308A*, respectively), and hence the meta-analyses were repeated after excluding the study (Table 4) and by using a random effects model (Additional file 1: Table S1). There was a lack of association between JIA and either *TNFA* variant when we included all studies; however, after excluding the Ozen et al. study, we identified an association between *TNFA-238A* and JIA (OR 0.69, 95% CI [0.52, 0.91], *p* = 0.0086) (Table 4). We observed no association between *TNFA-308A* and JIA (Table 4, Figure 1).

## Discussion

JIA is a complex trait believed to be influenced by both genetic and environmental factors [1]. Convincing associations between polymorphisms in the genes encoding the human leukocyte antigens (HLA) and JIA have been reported in multiple cohorts. *HLA-DR* is estimated to account for only ~17% of susceptibility to JIA, suggesting that non-HLA loci contribute substantially to JIA susceptibility [36]. To date, a few non-HLA variants have been demonstrated to have replicable associations. A comprehensive review of non-HLA associations suggested that most studies are underpowered and very few positive associations are replicated [2]. A handful of genes, including *PTPN22, TNFAIP3, STAT4, PTPN2,* and *CCR5,* have shown replicated associations [5,6,22,37], but many other associations have not been formally replicated. One strategy to improve power and develop a more accurate estimate of effect size in genetic associations is to perform meta-analysis of published studies to validate previous associations, as we did in this study. Our meta-analysis, with 3200 cases with JIA and over 5000 controls confirms a statistically significant association between *PTPN22* and JIA.

The gene *TNFA,* which encodes the proinflammatory cytokine TNF-α, is located in the MHC region on chromosome 6 and has been implicated in susceptibility to a number of rheumatic diseases, including rheumatoid arthritis. *TNFA* variants have been investigated for an association with JIA in a variety of studies. While associations between microsatellite polymorphisms in *TNFA* and different subtypes of JIA have been reported in various studies [13-15], investigations of two functional SNPs have yielded mixed results. We were able to find an association between the *G-238A* variant and oligoarticular JIA. The meta-analysis also suggests an association. Since the *TNFA* locus is in the MHC region, the associations observed could reflect linkage disequilibrium with HLA variants.

Our combined JIA cohort, while larger than several previously published cohorts, was still underpowered to detect small effects. For our primary analyses, we analyzed the JIA cohort as a whole, given the recent demonstration that clinically distinct autoimmune disorders share common susceptibility loci [38-40]. Since JIA is a collection of heterogeneous subtypes, we also performed stratified analyses of JIA sub-phenotypes in our data, but our power to detect associations with some of the less common JIA categories was low. Only one other study, Hinks et al., provided genotype data by category [10]. In a pooled analysis with their data, the importance of analysis by categories was emphasized; the strongest associations we found were in JIA subphenotypes, rather than the global JIA phenotype. Analyzing by subphenotype therefore appears to be a valuable procedure, as it reduces the negative impact of phenotypic and genetic heterogeneity.

A limitation with all candidate gene studies is the potential of population stratification. While we attempted to minimize the effects of stratification by selecting only self-reporting European ancestry for our cohort and including only studies from Northern European samples in our meta-analysis, we acknowledge that this effect could perhaps influence our findings. We were also concerned about site heterogeneity in our study, since controls were obtained from Utah, while cases were obtained from both Utah and Georgia. Therefore, we re-ran our analyses with only Utah cases (N = 432) and controls (N = 750). Although the standard deviation increased with decreasing sample sizes, point estimates remained approximately identical for all analyses, indicating that between -site heterogeneity does not to appear to be a concern in our study. Results of these analyses can be seen in Additional file 2: Table S2 and Additional file 3: Table S3.

Other meta-analyses between JIA and these variants have been published recently [41,42]. Lee et al. found no association between JIA and *TNFA G-238A* and *TNFA G-308A* variants among European subjects [41]. In contrast, our results do support an association between *TNFA G-238A* and JIA, and in particular oligoarticular JIA (significant after correction for multiple testing). Our meta-analysis also supported an association between this variant and JIA. As previously reported by Lee et al., we also did not find an association between JIA and *TNFA G-308A*[41]. Based on their meta-analysis, Lee et al. concluded there was association between *PTPN22 C1858T* and *MIF C-173G* variants [42]. While we confirmed the association between *PTPN22 C1858T* and JIA, we did not confirm the association with the *MIF C-173G* variant. There are several possible explanations for the discrepancies observed. First there might be true differences between the different populations being evaluated. Second, by the addition of almost 1400 subjects, our meta-analysis benefited from improved power. Third, the studies included in the meta-analyses varied somewhat. For instance, Lee et al. chose to include a study by Hohler et al., which investigated an association between TNFA G-238A and juvenile psoriatic arthritis and psoriasis [43]. We did not include the study by Hohler et al in our meta-analysis, since the subjects in their study were all adults, with “juvenile onset” having been defined as onset before the age of 40. In addition other subjects in that study had psoriasis without arthritis. Thus we felt their inclusion of these subjects in a meta-analysis of JIA was inappropriate. Finally, it should be noted that the minor allele frequencies extracted by Lee et al. for *PTPN22 C1858T* from the study by Thompson et al [6]. were substantially different than the actual frequencies reported in the original paper. Whereas Thompson et al. reported the case/control MAF for *PTPN22 C1858T* to be 0.143/0.094 and 0.149/0.095 for the initial and replication cohorts respectively (Tables 2 and 3 in Thompson et al. [6]), Lee et al. report these to be 0.249/0.193 and 0.252/0.204 for the same cohorts (Table 1, Lee et al. [42]).

## Conclusions

In conclusion, our study provides convincing, replicated evidence that *PTPN22* is associated with JIA. Our study also demonstrates the increased power of meta-analysis, and demonstrates that the *PTPN22 C1858T* variant is particularly associated with RF-negative, RF-positive and oligoarticular JIA categories, but not with ERA or systemic JIA. *PTPN22* C1858T variant has been identified as an underlying risk factor for several different autoimmune phenotypes [38,44,45]. Our finding supports the notion that clinically distinct autoimmune phenotypes can share common susceptibility factors, offering a potential target for further research and possible therapy. Our study also demonstrates an association between oligoarticular JIA and *TNFA-238A,* supporting future investigations of the TNF-α pathway in JIA.

## Competing interests

The authors declare that they have no competing interests.

## Authors’ contributions

MK, MRP, AW, MRB carried out the DNA extraction and genotyping of the subjects. LP recruited participants and organized participant data. SAH, KRS, LBV, JFB recruited subjects and supervised phenotypic data collection. KAB, MPE, KNC, SLG, and LBJ participated in the analysis of data and interpretation, and helped draft the manuscript. SP conceived of the study, participated in its design and coordination, recruited participants, and helped draft the manuscript. All authors read and approved the final manuscript.

## Supplementary Material

Additional file 1: Table S1 Meta-analyses performed allowing for random study effects. Click here for file

Additional file 2: Table S2 Case-control analysis of *PTPN22*, *TNFA* and *MIF* variants and JIA, including only Utah samples in analyses. Click here for file

Additional file 3: Table S3 Results of case-control association of PTPN22, MIF and TNFA variants among JIA sub-phenotypes, including only Utah samples in analyses. Click here for file
