# Supplementary material for: Meta-analysis confirms association between TNFA-G238A variant and JIA, and between PTPN22-C1858T variant and oligoarticular, RF-polyarticular and RF-positive polyarticular JIA
Source: Pediatr Rheumatol Online J. 2013 Oct 25;11:40. doi: 10.1186/1546-0096-11-40 (PMC3874734; doi:10.1186/1546-0096-11-40)
Supplement: Additional file 1: Table S1 — Meta-analyses performed allowing for random study effects. [file 1546-0096-11-40-S1.doc]

Supplementary Table 1: Meta-analyses performed allowing for random study effects

|  |  |  |  |
| --- | --- | --- | --- |
| Variant | OR (95% CI) | *p*-Value | Data Sources |
| PTPN22 C1858T | **1.44 (1.28, 1.62)** | **<0.0001** | 4-7, 18,19, 29 |
| MIF G-173C | **1.31 (1.04, 1.66)** | **0.0222** | 9, 13, 16 |
| TNFA G-238A | 0.83 (0.60, 1.14) | 0.2445 | 9, 11, 20, 22, 23 |
| TNFA G-308A | 1.01 (0.86, 1.41) | 0.4622 | 9, 11, 12, 20-23 |

Supplementary Table 1. Odds ratios, 95% confidence intervals, and *p*-values of pooled data for SNPs in PTPN22, MIF, TNFa238, and TNFa308. An unconditional generalized linear mixed-effects model with random study effects was used.
