# Supplementary material for: Meta-analysis confirms association between TNFA-G238A variant and JIA, and between PTPN22-C1858T variant and oligoarticular, RF-polyarticular and RF-positive polyarticular JIA
Source: Pediatr Rheumatol Online J. 2013 Oct 25;11:40. doi: 10.1186/1546-0096-11-40 (PMC3874734; doi:10.1186/1546-0096-11-40)
Supplement: Additional file 2: Table S2 — Case-control analysis of PTPN22, TNFA and MIF variants and JIA, including only Utah samples in analyses. [file 1546-0096-11-40-S2.doc]

Supplementary Table 2: Case-control analysis of *PTPN22*, *TNFA* and *MIF* variants and JIA, including only Utah samples in analyses

|  | Cases | | Controls | |  |  |
| --- | --- | --- | --- | --- | --- | --- |
| Variant | # Cases | MAF | # Controls | MAF | OR (95 % CI) | P |
| PTPN22 C1858T | 427 | 0.14 | 733 | 0.11 | **1.39 (1.08, 1.79)** | **0.01** |
| MIFG-173C | 425 | 0.19 | 742 | 0.17 | 1.11(0.89, 1.37) | 0.36 |
| TNFA G-238A | 427 | 0.04 | 749 | 0.06 | 0.70 (0.46, 1.04) | 0.09 |
| TNFA G-308A | 422 | 0.14 | 729 | 0.17 | **0.78 (0.61, 0.99)** | **0.04** |
