# Supplementary material for: Meta-analysis confirms association between TNFA-G238A variant and JIA, and between PTPN22-C1858T variant and oligoarticular, RF-polyarticular and RF-positive polyarticular JIA
Source: Pediatr Rheumatol Online J. 2013 Oct 25;11:40. doi: 10.1186/1546-0096-11-40 (PMC3874734; doi:10.1186/1546-0096-11-40)
Supplement: Additional file 3: Table S3 — Results of case-control association of PTPN22, MIF and TNFA variants among JIA sub-phenotypes, including only Utah samples in analyses. [file 1546-0096-11-40-S3.doc]

Supplementary Table 3: Results of case-control association of PTPN22, MIF and TNFA variants among JIA sub-phenotypes, including only Utah samples in analyses

|  | PTPN22 | | MIF | | TNFA G-238A | | TNFA G-308A | |
| --- | --- | --- | --- | --- | --- | --- | --- | --- |
| Category | # Cases | OR (95% CI) | # Cases | OR (95% CI) | # Cases | OR (95% CI) | # Cases | OR (95% CI) |
| Systemic | 33 | 1.33 (0.61, 2.54) | 32 | 1.39 (0.73, 2.49) | 33 | 2.03 (0.82, 4.38) | 33 | **0.24 (0.06, 0.66)^4^** |
| RF-Positive | 35 | **1.96 (1.04, 3.48)^1^** | 34 | 1.23 (0.67, 2.16) | 35 | 0.25 (0.01, 1.15) | 34 | 0.91 (0.45, 1.69) |
| RF-Negative | 95 | 1.08 (0.67, 1.68) | 96 | 0.94 (0.62, 1.39) | 97 | 1.33 (0.71, 2.32) | 95 | 0.79 (0.50, 1.02) |
| ERA | 32 | 1.45 (0.65, 2.88) | 32 | 1.31 (0.67, 2.40) | 32 | 0.82 (0.19, 2.33) | 32 | 0.45 (0.15, 1.02) |
| Oligoarticular | 203 | **1.40 (1.01, 1.92)^2^** | 203 | 1.08 (0.81, 1.42) | 203 | **0.30 (0.12, 0.60)^3^** | 202 | 0.88 (0.64, 1.18) |

1: p=0.03; 2: p=0.04; 3: p=0.002; 4: p=0.02
